# Application of artificial intelligence in echocardiography from 2009 to 2024: a bibliometric analysis

**DOI:** 10.3389/fmed.2025.1587364

**Published:** 2025-07-29

**Authors:** Yangli Liu, Lei Zhao, Bin Tu, Jie Wang, Yaqun He, Rufang Jiang, Xiaofeng Wu, Wen Wen, Jian Liu

**Affiliations:** Department of Ultrasound, Clinical Medical College, The First Affiliated Hospital of Chengdu Medical College, Chengdu, China

**Keywords:** echocardiography, artificial intelligence, bibliometric analysis, hot spots, visualization

## Abstract

**Background:**

Echocardiography is a cornerstone in the clinical diagnosis of cardiovascular diseases, providing critical insights into cardiac structure and function. Over recent years, artificial intelligence (AI) has emerged as a transformative adjunct to traditional echocardiographic techniques, enhancing diagnostic accuracy through innovations such as automatic view labeling, advanced image segmentation, and predictive disease modeling. The objective of this study is to explore the current status and prevailing research trends in this field from 2009 to 2024 through bibliometric analysis and to forecast future developmental trajectories.

**Methods:**

We selected the Science Citation Index Expanded (SCI-Expanded) from the Web of Science Core Collection (WOSCC) as our primary data source and conducted a comprehensive search encompassing all articles and reviews published between 2009 and 2024 and used the online analysis platform of bibliometrics, CiteSpace and VOSviewer software to analyze countries/regions, institutions, authors, keywords, and references, used Microsoft Excel 2021 to visualize the trends of the number of articles published by year.

**Results:**

Between 2009 and 2024, a total of 3,411 publications on AI applications in echocardiography were identified, including 3,000 articles (87.9%) and 411 reviews (12.1%), contributed by researchers from 100 countries/regions. China and the USA were the leading contributors in terms of publication volume. Notably, institutions such as Shanghai Jiaotong University demonstrated strong research productivity and international collaboration. *Journal of the American College of Cardiology* ranked among the most influential journals in this domain. Keyword analysis revealed that terms such as “artificial intelligence,” “machine learning,” “deep learning,” and “echocardiography” are central research hotspots, indicating emerging trends in the field and the potential to evolve into major areas of future investigation.

**Conclusion:**

Over the past decade, the integration of AI with echocardiography has become increasingly sophisticated. This study highlights the critical contributions of AI applications in echocardiography to the progression of the field and offers valuable insights for researchers embarking on future investigations.

## 1 Introduction

Echocardiography is one of the most important tools for assessing the structure and function of the heart, providing important information on ventricular wall motion, valve status, chamber size and hemodynamics. Consequently, it plays an indispensable role in the diagnosis and treatment of most cardiovascular diseases (1). The incidence of cardiovascular diseases has gradually increased with the improvement of living standards, and as a result, new techniques of echocardiography have been updated, such as three-dimensional echocardiography, speckle tracking, and semiautomated analysis. However, compared with other modalities, echocardiography is often affected by inter-observer variability and is strongly dependent on the level of experience (1). The limitations of echocardiography are evidently more prominent in the increasing demand and complexity, these include multiple measurements increasing the complexity of duration and subjectivity of the user, complex analyses during assessment, and high standards for personalized assessment (2). It is imperative to find ways to reduce inter-examiner variability in echocardiography to improve efficiency and reduce acquisition time.

With the rapid advancement of information and medical technology, artificial intelligence (AI) has become a major focus in the medical field, particularly in echocardiography, where it addresses inconsistencies and inter-and intra-observer variability in image acquisition and measurement processes (3). In fact, the integration of echocardiography and AI is not an entirely new topic. An earlier example of the application of echocardiography in conjunction with machine learning dates back to 1978, when Fourier analysis was used in M-mode ultrasound to assess the waveform of the anterior mitral valve leaflet. Studies have confirmed the significant impact of this method on the adjunctive diagnosis of mitral valve prolapse (4). In some cases, deep learning models now outperform humans in image recognition and have been used to develop fully automated echocardiography interpretation programs, including view recognition, image segmentation, quantification of structure and function, and disease detection (5). In recent years, AI applications in echocardiography have gained significant attention, although challenges remain, such as insufficient standardization, poor robustness, and limited generalization of models in clinical applications (6). Bibliometrics is a well-known effective method for studying influential authors, journals, or countries/regions, building collaborative networks, and identifying research hotspots in specific fields (7, 8). This study aims to explore the current research status and trends from 2009 to 2024 through bibliometric analysis and to predict future developments in this field.

## 2 Methods

### 2.1 Data source

Scopus and the Web of Science (WOS) are the primary databases used for bibliometric research (9). The data for this study were retrieved from the Web of Science Core Collection (WoSCC) SCI-Expanded database, a widely used platform known for its rigorous indexing and comprehensive coverage of high-impact journals (10). While Scopus offers broader journal coverage including conference proceedings and regional publications (11), WoSCC is favored for its standardized citation metrics and data reliability, which are crucial for bibliometric analysis. Combining these databases can enhance comprehensiveness; however, this study focused on WoSCC to ensure data consistency and accuracy.

### 2.2 Data collection

Data from 2009 to 2024 in the field of AI and echocardiography were retrieved on 16 October 2024. The retrieval strategy for this study was as follows: TS = (artificial intelligence OR machine learning OR data learning OR deep learning OR intelligent learning OR supervised learning OR unsupervised learning OR reinforcement learning OR neural network OR Bayes network OR feature learning OR feature selection OR support vector machine OR random forest OR semantic segmentation OR image segmentation OR k-nearest neighbor) AND TS = (echocardiograph OR echocardiogram OR echocardiography OR (speckle tracking OR longitudinal strain OR left ventricular ejection fraction (LVEF) OR Simpson)) The data categories are restricted to “articles” and “reviews.”

### 2.3 Data analysis

Microsoft Excel 2021, CiteSpace and VOSviewer software (12) were used for descriptive statistical analysis of publications. Three different visualization maps with different meanings (network, density, and overlay visualization) and the total link strength (TLS) were generated by VOSviewer (version 1.6.20). TLS was used to quantitatively assess the links. Many nodes are connected by different lines. The links between the nodes indicated correlation between parameters (13). VOSviewer was used to visualize country/regional cooperation, author co-authorship, and keyword co-occurrence. In the visual map, different nodes represent authors, journals, keywords, etc.; the node size indicates the number or frequency; the thickness of the line represents the strength of the link; and the node colors represent different clusters or times. CiteSpace (6.2. R3) was used to analyze the included literature, including co-citation analysis of countries/regions and institutions, dual-map overlay of citations, timeline view, co-cited reference analysis, and references with the strongest citation bursts. Microsoft Excel 2021 was used for visualize the trends of the number of articles published by year and draw the polynomial fitted curves of annual publication. What’s more, the Charticulator website and the Scimago Graphica software was used to build a network of international cooperation between countries.

## 3 Results

### 3.1 Annual publications analysis

**Figure 1:**
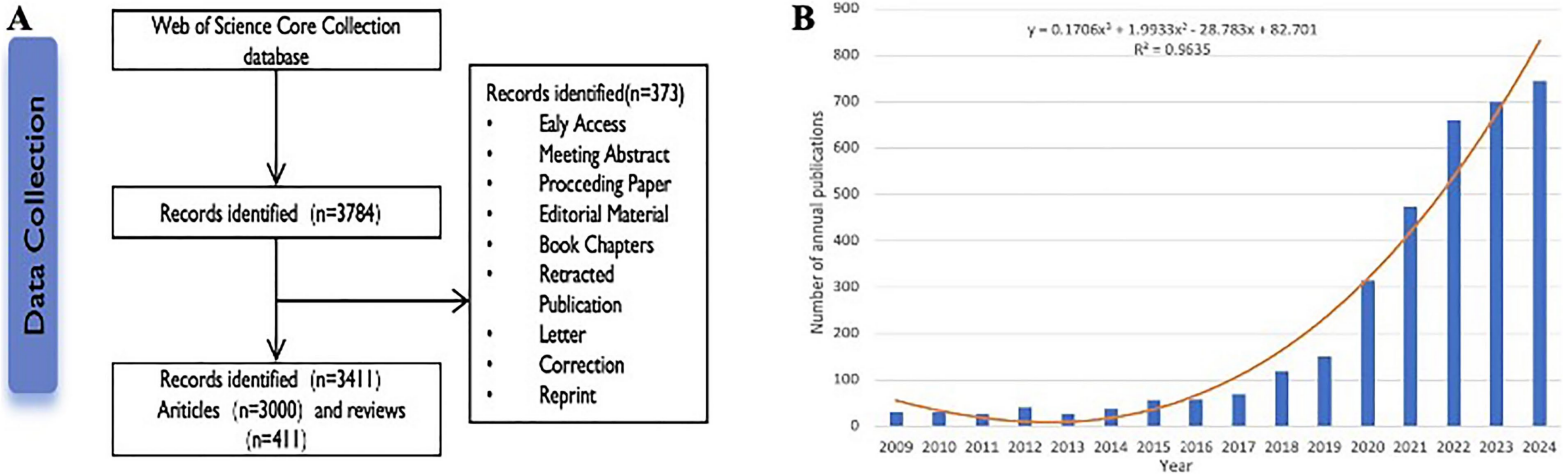
**(A)** Flowchart of literature selection; **(B)** Global publication growth trend from 2009 to 2024. Each entry shows the number of publications per year. The curves represent a curve fit to this trend.

As depicted in Figure 1A, between 1 January 2009, and 31 December 2024, 3,411 publications on application of AI in echocardiography were included. There were a total of 3,000 articles (87.9%) and 411 reviews (12.1%) from 20,331 scholars in 100 countries/regions published in 1,014 journals. As shown in Figure 1B, the number of publications on Application of AI in echocardiography has increased exponentially in the last 5 years, with a significant correlation coefficient (R^2^ = 0.9635) between the two variables. The number of publications has been consistently low flat until 2018. The publication volume reached its peak in 2024 (745, 21.8%). According to the current trend, it is hypothesized that this field will be a hotspot of interest in the future.

### 3.2 Countries analysis

**Figure 2:**
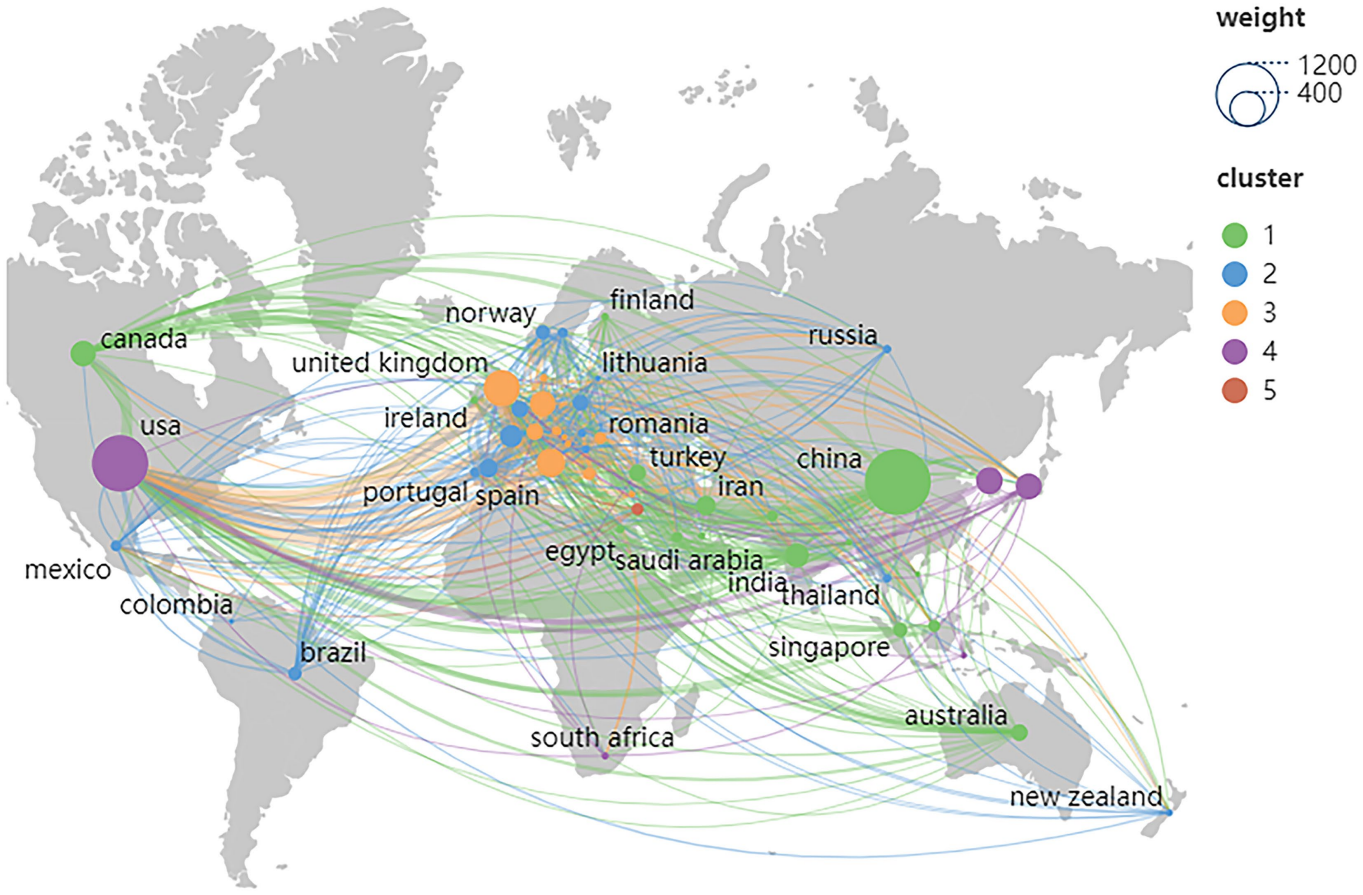
Geographical visualization of country cooperation.

From 2009 to 2024, a total of 100 countries or regions contributed to publications in the Web of Science. The 10 most productive countries/regions are listed in Table 1. It is worth noting that Application of AI in echocardiography is booming in China and the USA. China (1,198 publications and 13,784 citations) was the most productive country, followed by the USA (874 publications and 19,639 citations) and United Kingdom (365 publications and 8,139 citations). Although China ranks first in total publications, its average citation value (value = 11.51) is the ninth most productive among the 10 countries/regions, just ahead of India. TLS indicates the impact of country/region publications on other countries/regions involved in the study. The USA has the highest TLS (881 TLS), followed by the United Kingdom (568 TLS) and China (376 TLS). The geo-visualization map of collaboration between countries/regions is shown in Figure 2. The United States had the most cooperation with other countries worldwide, especially with Nordic countries.

**Table 1:** The 10 most productive countries/regions.

Rank	Country	Documents (*n*)	Citations	Average citations	TLS
1	China	1,198	13,784	11.51	376
2	USA	874	19,639	22.47	881
3	United Kingdom	365	8,139	22.29	568
4	Italy	223	3,613	16.2	419
5	South Korea	197	3,398	17.25	62
6	Japan	187	2,833	15.15	95
7	Canada	178	3,426	19.25	241
8	Germany	176	3,872	22	290
9	India	155	1756	11.32	184
10	Frence	131	2,583	19.72	199

TLS, total link strength.

**Figure 3:**
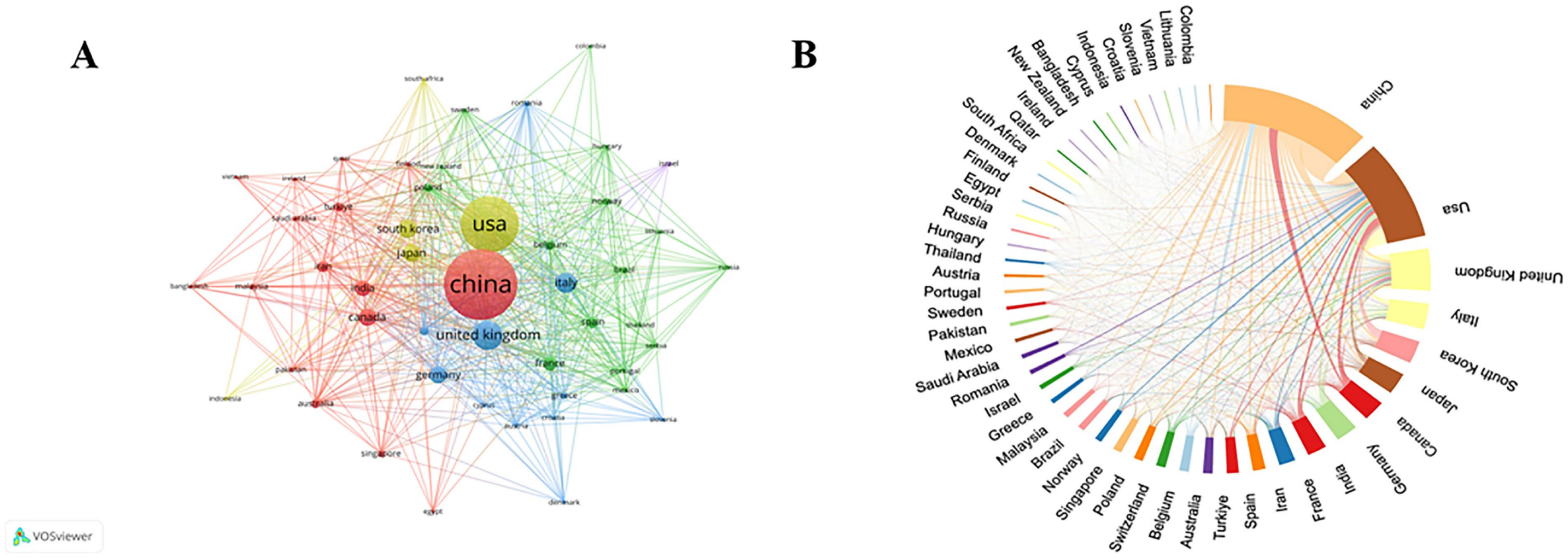
**(A)** The countries/regional co-authorship networks; **(B)** The international Co-operation Networks between Countries.

International cooperation between countries/regions is shown in Figure 3 using VOSviewer. with 50 countries/regions included in our analysis (Figure 3A). Each node in the network represents a country or region, and the node size represents the number of publications. The connections between nodes represent cooperation between countries. Figure 3B, produced through the Charticulator website, shows the analysis of co-authorship between countries or regions. The length of the nodes represents the number of publications, and the width of the connecting line indicates the frequency of collaboration between countries. China is the center of research on AI applications in echocardiography, with close collaboration with the USA and the United Kingdom.

### 3.3 Institutions analysis

A total of 5,013 institutions contributed to the publications on AI applications in echocardiography. The top 10 contributing 12.55% (629 publications) of the total paper. As shown in Table 2, The top 10 institutions are mainly located in China (*n* = 6) and among the top 10 institutions, China (401 publications) has nearly twice as many publications as all other countries (228 publications) combined. The institution with the most publications was Shanghai Jiaotong University (91 papers) followed by Sun Yat-sen University (78 papers) and the Mayo Clinic (74 papers), although the Mayo Clinic was in third place in terms of publications on this study, it had the highest number of citations (2,075 citations) and average citation value (value = 28.04) of all institutions.

**Table 2:** The 10 most productive institutions.

Rank	Institution	Country	Documents (*n*)	Citations	Average citations	TLS
1	Shanghai Jiao Tong Univ	China	91	1,176	12.92	198
2	Sun Yat Sen Univ	China	78	1,373	17.6	197
3	Mayo Clin	USA	74	2075	28.04	122
4	Chinese Acad Sci	China	70	1,277	18.24	144
5	Yongsei Univ	South Korea	61	927	15.19	69
6	Fudan Univ	China	60	1,338	22.3	137
7	Zhejiang Univ	China	57	648	11.36	132
8	Univ Oxford	England	47	823	17.51	118
9	Harvard Med Sch	USA	46	1,087	23.63	184
10	Southern Med Univ	China	45	722	16.04	90

TLS, total link strength.

**Figure 4:**
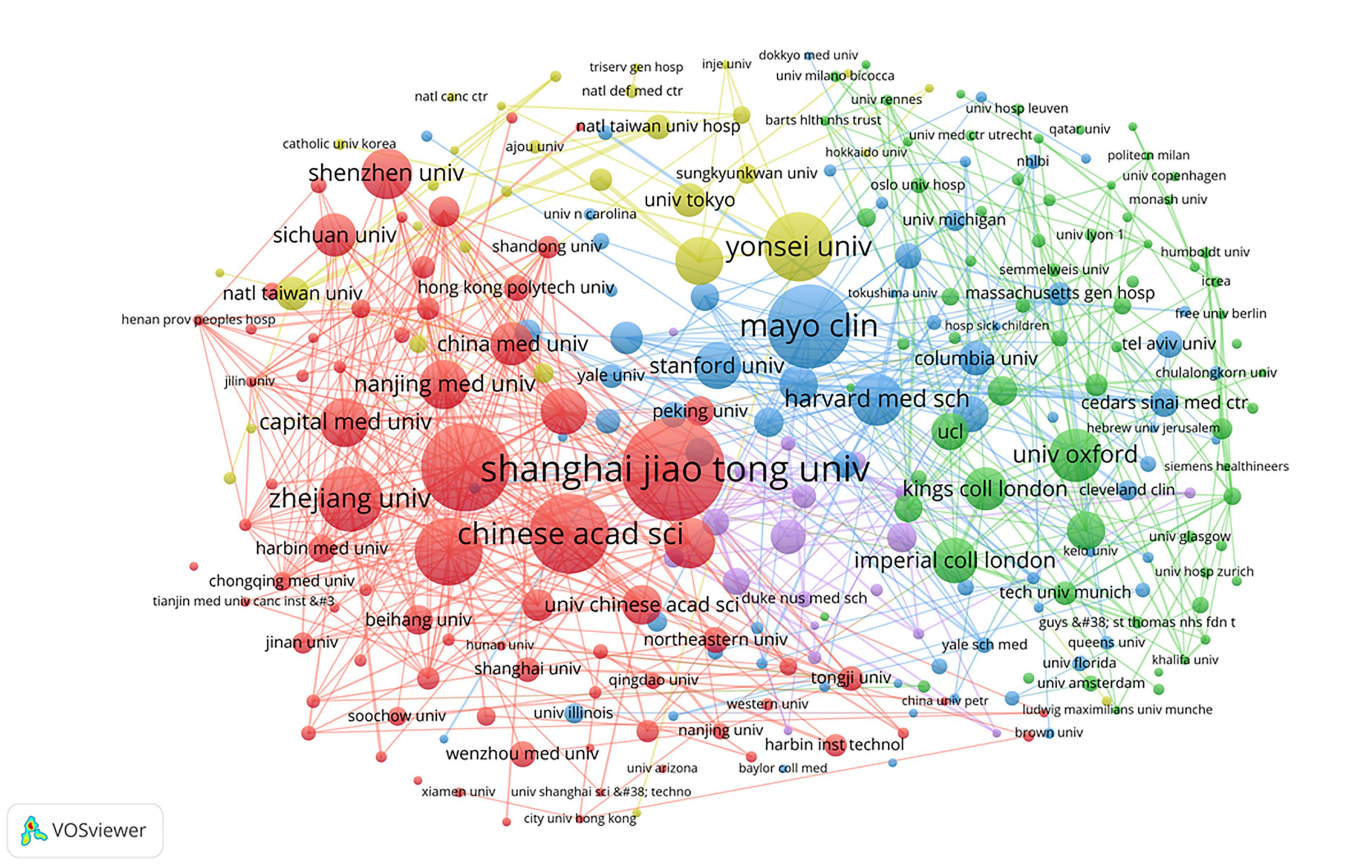
Co-operation network between institutions.

Figure 4 shows the construction of a visualization map of the institutional collaboration network through the VOSviewer software with a minimal productivity of 8 publications (*n* = 265). Each node represents the institution, the size of the node represents the number of published papers by the institution, and the connective lines between the nodes represents the cooperation between the institutions. More links indicate closer cooperation between the institutions.

### 3.4 Authors and co-cited authors analysis

Between 2009 and 2024, a total of 20,331 authors and 71,937 co-cited authors made contributions to the field of AI in echocardiography. Table 3 lists the top 10 most productive authors. There are 5 authors from the USA, 3 of whom are all from the Mayo Clinic. The remaining 5 authors are from China, Australia, and Norway. Yang, Xin (26 papers and 381 citations) from Shenzhen University in China is the most productive author from 2009 to 2024, followed by Friedman, Paul A (25 papers and 1,373 citations) and Lopez-Jimenez, Francisco (25 papers and 1,355 citations) both of Mayo Clinic. however, although Yang, Xin has the most publications, he has the lowest citations. In terms of average citations, the top three were all from the Mayo Clinic, and therefore, the Mayo Clinic is an authority in the field of AI applications in echocardiography.

**Table 3:** The 10 most productive authors.

Rank	Author	Institution (country)	Documents (*n*)	Citations	Average citations
1	Yang, Xin	Shenzhen University (China)	26	381	14.65
2	Friedman, Paul A	Mayo Clinic (USA)	25	1,373	54.92
3	Lopez-jimenez, Francisco	Mayo Clinic (USA)	25	1,355	54.2
4	Acharya, Rajendra U	University of Southern Queensland (Australia)	25	589	23.56
5	Sengupta, Partho P	West Virginia of University (USA)	23	928	40.35
6	Noseworthy, peter A	Mayo Clinic (USA)	20	1,335	66.75
7	Ni, Dong	Zhejiang University (China)	20	288	14.4
8	Wang, Wei	Southern Medical University (China)	20	472	23.6
9	Lovstakken, Lasse	Norwegian University Science and Technology (Norway)	18	565	31.39
10	Ouyang, David	Cedars Sinai Medical Center (USA)	17	875	51.47

**Figure 5:**
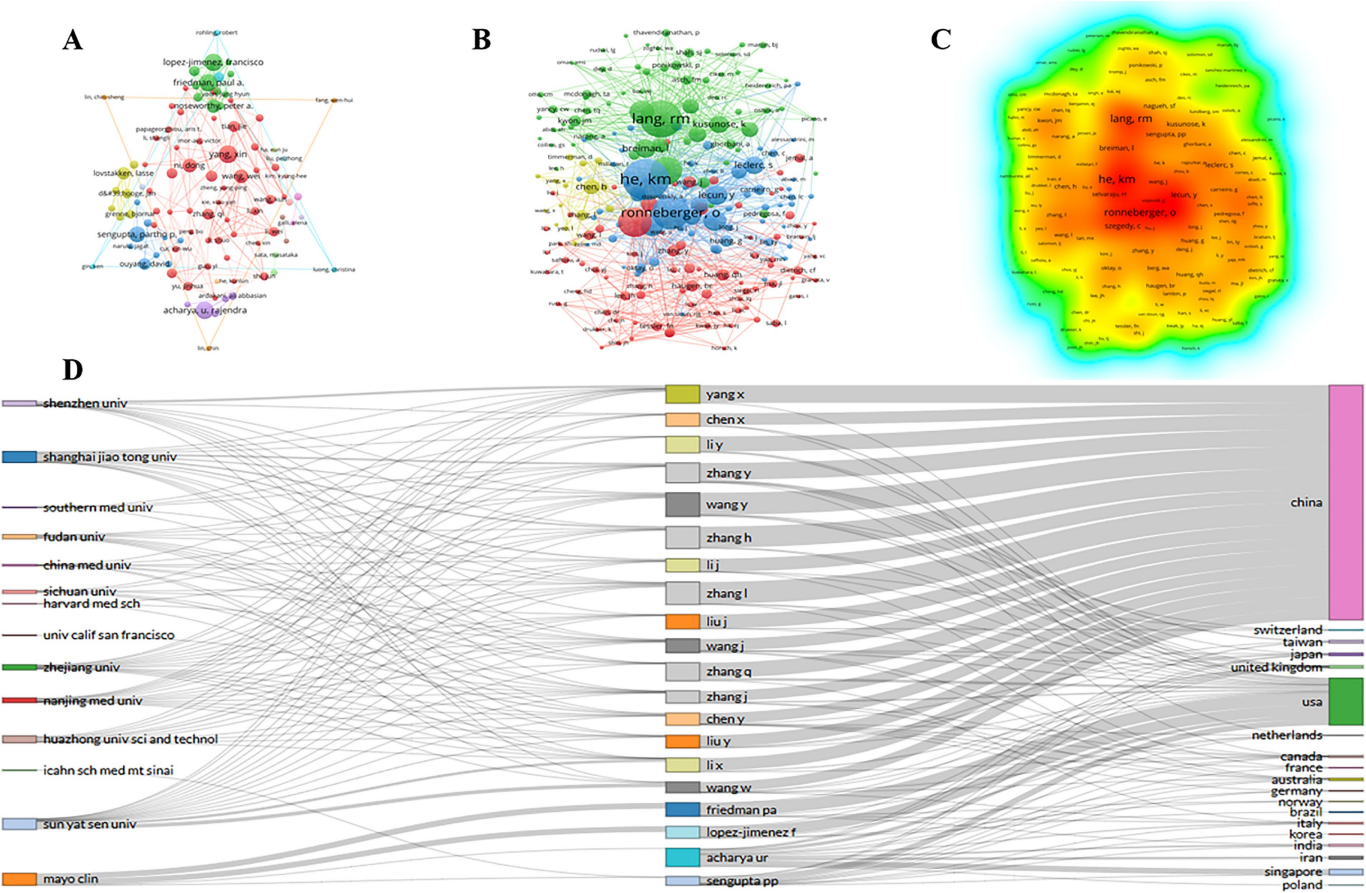
**(A)** Co-operation network between authors; **(B)** Co-operation network between co-cited authors; **(C)** VOSviewer density visualization of the co-cited authors; **(D)** Three-field diagram represents the incoming and outgoing flows between top authors, institutions and research contributors over the last 15 years.

The contribution of authors with a minimum productivity of 8 publications (*n* = 50) were visualized by VOSviewer software, as shown in Figure 5A. Each node represents an author, the size of the node represents the number of papers, and the lines represent the collaboration between authors.

Co-cited authors mean that the authors are cited together (14). Supplementary Table S1 lists the top 10 most citations co-cited authors. Similarly, there are 5 authors from the USA, and the remaining 5 are from China, Australia and Germany. He Kangmin (411 citations and 5,545 TLS) from University of Chinese Academy of Sciences in China, followed by Lang, R.M (369 citations and 4,249 TLS) from University of Chicago in USA and Ronneberger, Olaf (330 citations and 4,489 TLS) from University of Freiburg in Germany. The co-operation network between co-cited authors with a minimal number of 50 citations (*n* = 152) were shown in Figure 5B. Moreover, Figure 5C shows a density map of the co-cited authors. The deeper color (red) indicates a higher number of citations.

In order to observe the outgoing and incoming flows between countries, institutions and authors contributing to research from 2009 to 2024, We used the R programming language to create a three-field plot, also known as a “Sankey diagram.” We selected “Institution” on the left, “Authors” in the middle field, and “Country” on the right to draw the network relationships. The number of items was limited to 20. The American authors are mainly from the Mayo Clinic, while the Chinese authors are mostly from different institutions (Figure 5D).

### 3.5 Journals analysis

Between 2009 and 2024, a total of 1,014 journals published 3,411 papers and 19,621 co-cited papers in the field of AI in echocardiography. The journals were ranked according to the number of articles published. The top 10 journals in terms of publications are listed in Table 4. *Ultrasound in Medicine and Biology* (89 publications, 1,248 citations and 257 TLS) is the most published article, followed by *Diagnostics* (88 publications, 435 citations and 261 TLS) and *Frontiers in Cardiovascular Medicine* (85 publications, 445 citations and 260 TLS). Although *Medical Image Analysis* (49 publications, 1,402 citations and 273 TLS) has the least number of published articles, it has the most citations and TLS. The 10 most citations co-cited journals are shown in Supplementary Table S2. The top three were *Journal of the American College of Cardiology* (2,721 citations, 113,572 TLS and impact factor (IF) = 19.89), *Circulation* (2,642 citations, 105,746 TLS and IF = 23.05) and *IEEE Transaction on Medical Imaging* (2,244 citations, 78,290 TLS and IF = 8.9).

**Table 4:** The 10 most productive journals.

Rank	Journal	Citations	TLS	Impact factor (2024)	JCR
1	Journal of the American College Of Cardiology	2,721	113,572	19.89	Q1
2	Circulation	2,642	105,746	23.05	Q1
3	IEEE Transaction on Medical Imaging	2,244	78,290	8.9	Q1
4	Lecture Notes in Computer Science	2,138	70,903	0.32	Q4
5	Radiology	2015	81,118	12.1	Q1
6	Journal Of The American Society of Echocardiograph	1868	69,741	5.4	Q1
7	Proceedings. IEEE Computer Society Conference on Computer Vision and Pattern Recognition	1800	54,229	21.76	Q1
8	European Heart Journal	1,645	72,280	20.21	Q1
9	Ultrasound in Medicine and Biology	1,634	60,821	2.4	Q2
10	JACC-Cardiovascular Imaging	1,506	70,026	12.8	Q1

TLS, total link strength.

**Figure 6:**
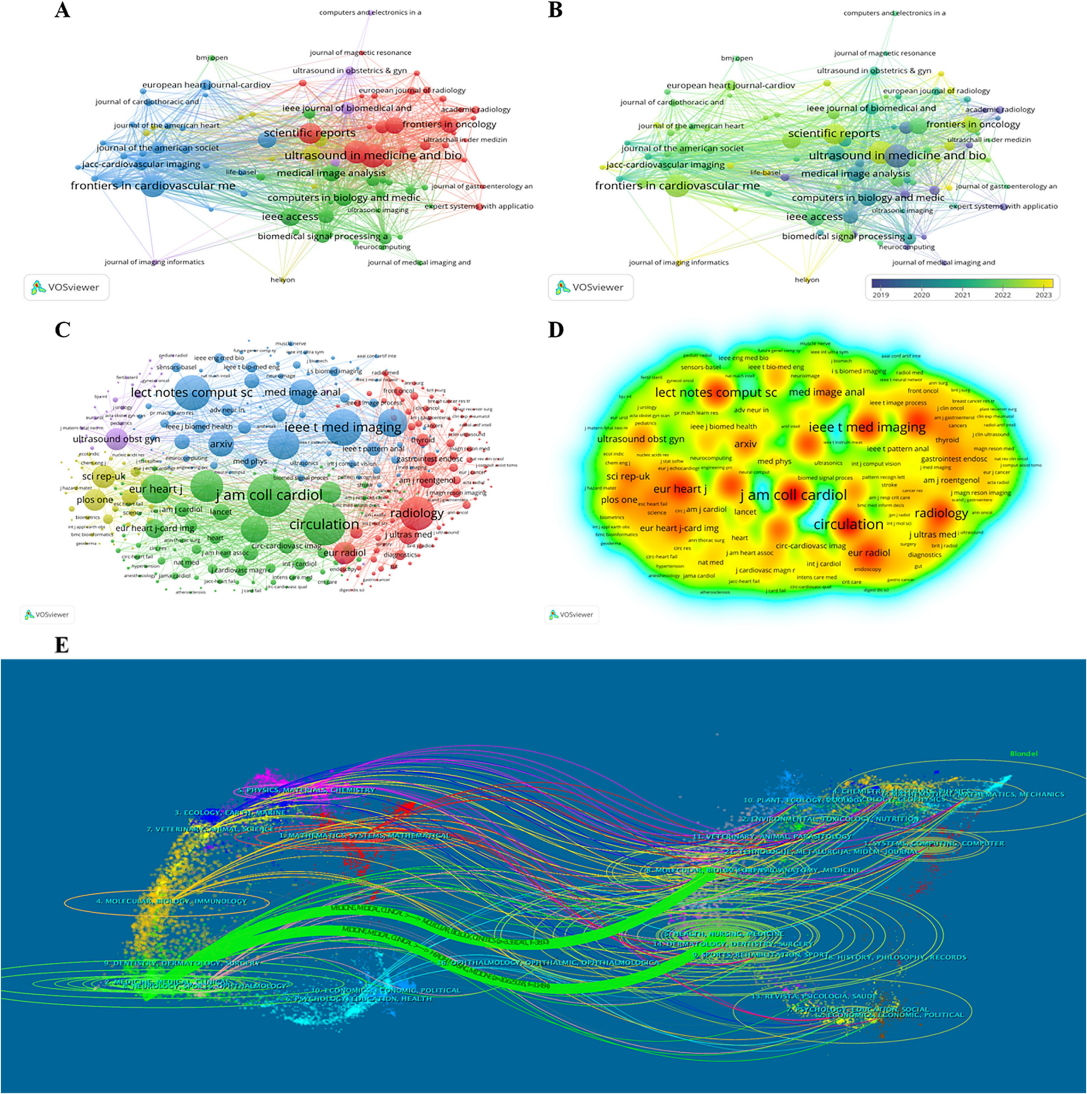
**(A)** Co-operation network between journals; **(B)** The time-overlay map of the cooperation network between journals; **(C)** Co-operation network between co-cited journals; **(D)** VOSviewer density visualization of the co-cited journals; **(E)** Dual-map overlay of journals related to the application of artificial intelligence in echocardiography.

The journals with a minimum productivity of 8 publications (*n* = 96) were visualized by VOSviewer software, as shown in Figure 6A. Each node represents a journal, the connectivity between the nodes represents the collaboration between the journals, and the size of the nodes represents the number of publications of the journals in the field of AI in echocardiography applications. Their collaborations are divided into 5 clusters and, we also analyzed to get a visualization of the overlay of the year (Figure 6B). It represents the time when the application field of AI in Echocardiography was published in the journal. The colder (blue) colors represent the more distant years, and the warmer (yellow) colors represent the more recent years. Similarly, Co-cited journals analysis was performed in Figure 6C, and the density visualization is shown in Figure 6D. The deeper color (red) indicates a higher number of citations.

Figure 6E displayed the dual-map overlay of journals using CiteSpace software. The left and right sides corresponded to the citing and cited journals, respectively. The labels represent the topics covered by the journals, and the colored curves reflect the citation paths (15). There were two main citation paths, indicating that articles in health/nursing/medical journals were frequently cited in articles in Molecular/Biological/Immunological journals and articles in Molecular/Biological/Genetic journals were frequently cited in articles in Medical/Medicine/Clinical journals. The vertical axis of the ellipse represents the volume of papers in the topic and the horizontal axis represents the number of authors. Thus, the number of articles and authors cited in health/nursing/medical journals is the highest.

### 3.6 Reference co-citation analysis and the strongest citation burst

We analyzed 109,029 cited references, of which the top 10 are shown in Table 5. Titled “U-Net: Convolutional Networks for Biomedical Image Segmentation” was published by Prof Ronneberger, Olaf in the journal Lecture Notes in Computer Science in 2015, and it is one of the most cited articles. Followed by titled “Deep Residual Learning for Image Recognition” was published by Prof He, Kangmin in the journal PROC CVPR IEEE in 2016 and “Recommendations for Cardiac Chamber Quantification by Echocardiography in Adults: An Update from the American Society of Echocardiography and the European Association of Cardiovascular Imaging” was published by Prof Lang, R.M in the journal European Heart Journal in 2015.

**Table 5:** The 10 most co-cited references.

Rank	Title	Journal	First author	Citations	Year
1	U-Net: Convolutional Networks for Biomedical Image Segmentation	Lecture Notes in Computer Science	Ronneberger, Olaf	306	2015
2	Deep Residual Learning for Image Recognition	Proceedings. IEEE Computer Society Conference on Computer Vision and Pattern Recognition	He, Kangmin	285	2016
3	Recommendations for Cardiac Chamber Quantification by Echocardiography in Adults: An Update from the American Society of Echocardiography and the European Association of Cardiovascular Imaging	European Heart Journal	Lang, R. M	198	2015
4	Fully Automated Echocardiogram Interpretation in Clinical Practice: Feasibility and Diagnostic Accuracy	Circulation	Zhang, J	191	2018
5	ImageNet Classification with Deep Convolutional Neural Networks	Communications of the Acm	Krizhevsky, Alex	153	2017
6	Video-based AI for beat-to-beat assessment of cardiac function	Nature	Ouyang, David	148	2020
7	Deep Learning for Segmentation Using an Open Large-Scale Dataset in 2D Echocardiography	IEEE Transaction on Medical Imaging	Leclerc, Sarah	135	2019
8	Deep learning	Nature	LeCun, Yann	118	2015
9	A survey on deep learning in medical image analysis	Medical Image Analysis	Litjens, Geert	113	2017
10	Fast and accurate view classification of echocardiograms using deep learning	NPJ Digital Medicine	Madani, Ali	105	2018

TLS, total link strength.

**Figure 7:**
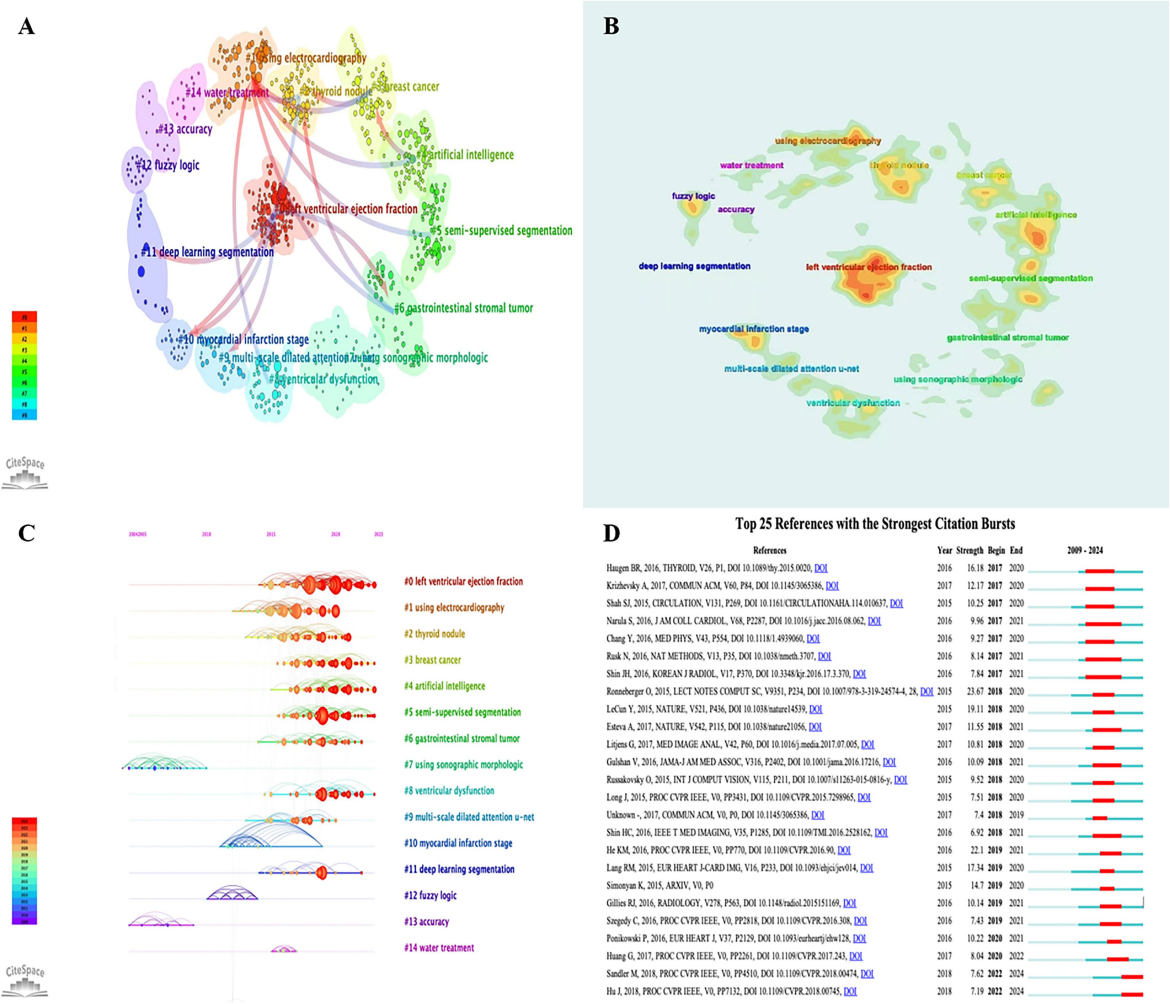
**(A)** Co-cited references visualization network and cluster; **(B)** Co-cited references density visualization networks and cluster; **(C)** Timeline plot of references’ co-cited analysis; **(D)** Top 25 references with citation bursts.

Reference co-citation analysis uses reference as the element of analysis to reflect the relationship between the reference by analyzing patterns and trends in co-citation (16). In CiteSpace the Q value of modularity is a measure of the significance of the association structure, when Q > 0.3 it means that the delineated association structure is significant. When the silhouette value (S) > 0.7 indicates that the clustering is efficiently convincing. Figure 7A intuitively represents that we clustered the research themes into 14 main clusters using CiteSpace software. From the results of the analysis the Q value is 0.8511 and the S value is 0.9296, indicating that the clustering effect and network homogeneity are reliable. The arrows indicate the evolution of this research theme. We also made a heat map about the 14 research topics using the latest version of the CiteSpace software (Figure 7B), which reflects the heat of each topic in the application of AI in echocardiography. Figure 7C shows a timeline view of the co-cited literature, reflecting the evolution of research hotspots over time. In this study # 0 LVEF is the largest cluster, showing the hot area of research on the application of AI in echocardiography. Followed by #1 using electrocardiography, #2 thyroid nodule, #3 breast cancer, #4 artificial intelligence, #5 semi-supervised segmentation, #6 gastrointestinal stromal tumor, #7 using sonographic morphologic, #8 ventricular dysfunction. Other major clusters include #9 muti-scale dilated attention u-net, #10 myocardial infarction stage, #11 deep learning segmentation, #12 fuzzy logic, #13 accuracy, #14 water treatment.

We also applied CiteSpace to analyze the top 25 references with the strongest citation bursts from 2009 to 2024 (Figure 7D). The blue line segments indicate the time intervals and the red line segments indicate the times of frequent references. The strongest citation burst has occurred in Ronneberger, O et al. in 2015, followed by LeCun, Y et al. in 2015 and Lang RM et al. in 2015.

### 3.7 Keyword analysis

Keywords are words or phrases carefully selected by authors to help readers and retrieval systems quickly understand the subject of an article and they play an important role in hot topics and trends. In the study according to the keyword co-occurrence analysis in VOSviewer 11,933 keywords were collected and we list the top 10 keywords in Table 6. The top three most frequently occurring keywords were “Deep Learning” (657 occurrences) “Machine Learning” (639 occurrences) and “Artificial Intelligence” (627 occurrences). respectively

which were consistent with our research theme. The other keywords for related topics include “Echocardiography,” “Ultrasonography,” “Ultrasound,” “Diagnosis,” “Classification,” “segmentation,” “management.”

**Table 6:** The 10 most occurrences keywords.

Rank	Keyword	Occurrences	TLS
1	Deep Learning	657	3,810
2	Machine Learning	639	3,854
3	Artificial Intelligence	627	3,880
4	Echocardiography	592	3,732
5	Ultrasonography	557	3,340
6	Ultrasound	512	3,035
7	Diagnosis	468	2,904
8	Classification	438	2,702
9	Segmentation	225	1,414
10	Management	212	1,470

TLS, total link strength.

**Figure 8:**
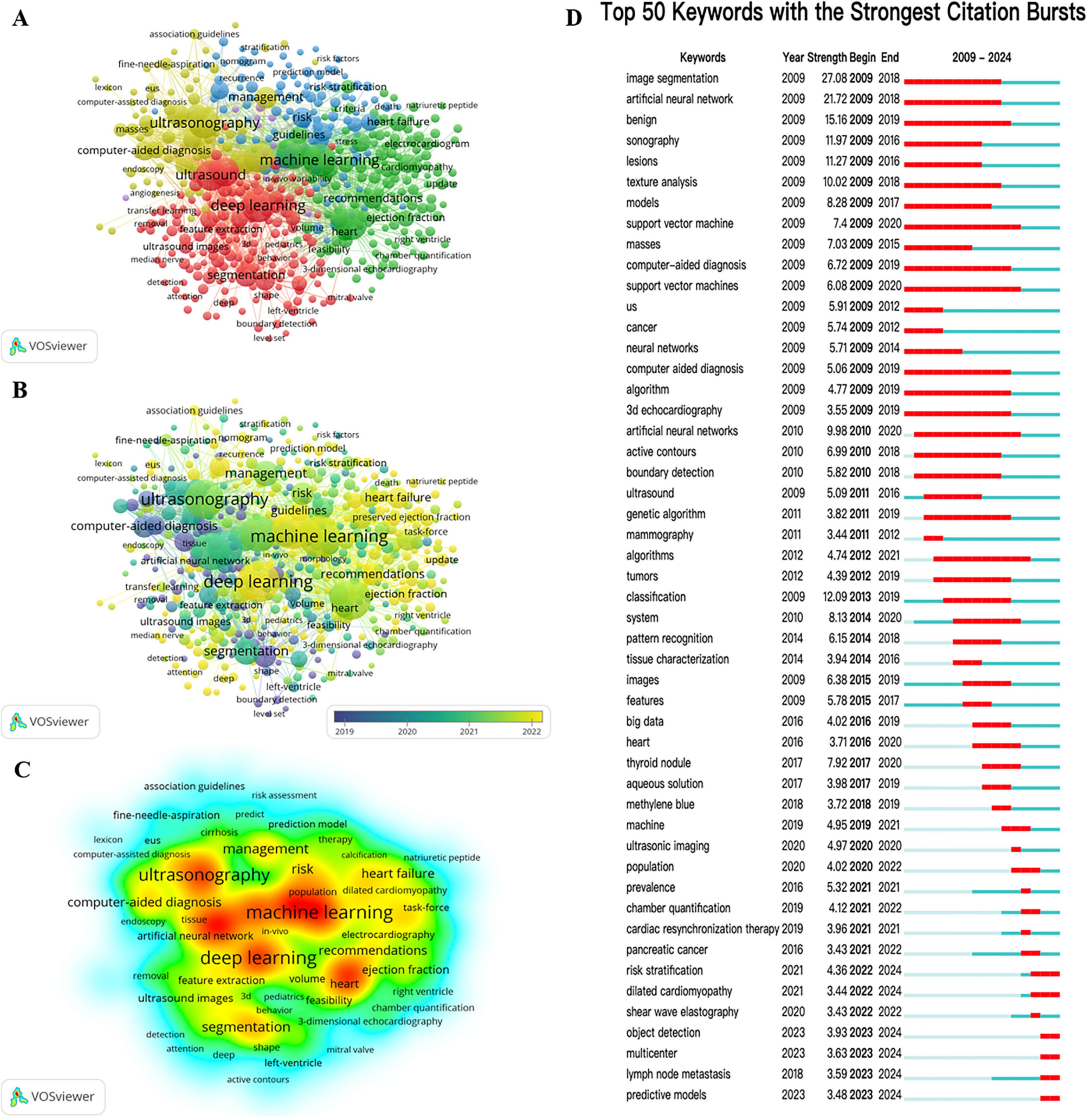
**(A)** VOSviewer cluster visualization of keywords; **(B)** VOSviewer density visualization of keywords; **(C)** Top 50 keywords with citation bursts; **(D)** Top 25 references with citation bursts.

The keywords with a minimum occurrence of 8 occurrences (*n* = 587) were visualized by VOSviewer software, as shown in Figure 8A. We also analyzed the visualization of the year overlay (Figure 8B) and the density visualization (Figure 8C). It represents the research trends and focus in the application field of AI in echocardiography. Using CiteSpace to capture burst keywords and construct timeline views to further explore emerging research hotspots associated with AI and echocardiography. As shown in Figure 8D. The blue line segments represent the time intervals and the red line segments indicate the times of frequent references. The keywords “Image segmentation” and “artificial neural networks” are the two keywords that have exploded with the highest burst intensity. They were also revealed to be the key research field for AI in echocardiography applications. In addition, the keywords such as “risk stratification”; “dilated cardiomyopathy”; “object detection” and “predictive models” have become the popular research recently.

## 4 Discussion

### 4.1 General information

This study analyzed 3,411 papers on the use of AI in echocardiography from 100 countries over the period from 2009 to 2024. We utilized the online bibliometric analysis platform, along with CiteSpace and VOSviewer software, to conduct bibliometric and visualization analyses. The research trends and hotspots in the field were examined by summarizing annual publications, countries/regions, authors, institutions, journals, and keywords.

The number of publications on the use of AI in echocardiography has surged rapidly since 2018, reaching a peak this year. This upward trend suggests that this field will attract significant interest in the future. China and the USA are the primary contributors, publishing more papers than any other countries/regions.

Although China leads in total publications, its average citation value ranks ninth among the top 10 most productive countries/regions, just ahead of India. In addition, more than half of the top 10 institutions in terms of publications are located in China. Furthermore, more than half of the top 10 institutions in terms of publications are based in China. Despite the Mayo Clinic in the USA ranking third in the number of papers in this study, it boasts the highest number of citations and the highest average citation value among all institutions, underscoring its authority and influence in this field.

Among the authors, Yang, Xin from Shenzhen University in China stands out as the most prolific, followed by Paul A. Friedman and Francisco Lopez-Jimenez, both affiliated with the Mayo Clinic. Notably, half of the top 10 most co-cited authors are from the USA, highlighting the country’s significant contributions to this field. Additionally, as we all know, selecting the right journals is an indispensable skill for researchers, and by analyzing the journals trends we assist researcher to select the right journals and thus contribute more to the field. The journal with the highest number of published articles is *Ultrasound in Medicine and Biology*, followed by *Diagnostics* and *Frontiers in Cardiovascular Medicine.* Although *Medical Image Analysis* has the lowest number of published articles, it has the highest number of citations and TLS, which suggests that the journal features many high-quality articles in this field. What’s more, the most cited journal are *Journal of the American College of Cardiology*, *Circulation* and *IEEE Transaction on Medical Imaging.*

### 4.2 Current research status

Echocardiography stands as a crucial tool in the clinical diagnosis of cardiovascular diseases, as indicated by numerous studies (6). Despite its critical role, echocardiography faces certain limitations, notably its reliance on the experience of the operator and variability in results between different observers. To address these significant challenges, researchers have suggested incorporating AI as a potential solution to enhance the accuracy and consistency of echocardiographic assessments. AI is a program that has tasks based on algorithms in an intelligent manner, mainly including machine learning and deep learning (17). Machine learning involves the development of computer algorithms designed to identify characteristic patterns between input data (such as images) and output results. These algorithms are then tested and validated on similar datasets to ensure their effectiveness. Examples include artificial neural networks, random forest algorithms and support vector machines (18, 19). Deep learning is a branch of machine learning that addresses problems through the use of multilayered neural networks. For examples stacked autoencoders, deep Boltzmann machines and convolutional neural networks, which are most commonly used in imaging medicine (20). Currently, the combination of AI techniques to measure cardiac volume, evaluate myocardial function and valve quantification have played an important role in clinical practice.

#### 4.2.1 Automatic quantitative evaluation of cardiac volume

Quantification of cardiac size and function is an important evaluation criterion of echocardiography. The evaluation of left ventricular (LV) function is a vital and standard component of diagnostic echocardiography. Indicators of LV systolic function include LVEF, LV volume, LV wall motion function, myocardial contractility, and global longitudinal strain (GLS) (6). LVEF is the most convenient and commonly used index to evaluate LV systolic function. The most common clinical measurements are endocardial curves traced in apical 4-chamber and 2-chamber views, and volume and EF calculated by the modified Simpson method (21, 22). However, challenges such as time consumption and poor consistency among observers, both within the same individual over time (intra-observer) and across different individuals (inter-observer), persist. To address these issues, advancements in AI technology have led to the development of AI-driven automatic 3D echocardiographic quantitative evaluation tools. These innovations have gained approval for clinical use, exemplified by the HeartModel algorithm in the Philips EPIQ series. This software displays an automated tracing of the endocardial borders of the LV and left atrium (LA) by 3D analysis (23, 24). This technology enables the simultaneous acquisition of LV end-diastolic and end-systolic volumes, LV volumes, and EF, all accomplished with minimal analysis time while ensuring high accuracy and reproducibility of results. Recently, GE has introduced 4D automated LV volumetric analysis and 4D automated LA volumetric analysis technologies, which also automatically measure LV and LA volumes and EF in three dimensions (17).

#### 4.2.2 Automated myocardial strain analysis

The European Society of Cardiovascular Imaging and the American Society of Echocardiography recommend the use of GLS as a complement to LVEF in the evaluation of left ventricular function (25). Strain refers to the myocardium’s capacity to deform under tension, providing insight into myocardial contractile function. GLS has an extremely important role in the assessment of overall left ventricular systolic function as recommended by the guidelines for quantitative cardiac chambers in adult echocardiography (25). Currently, myocardial strain is usually detected using a two-dimensional speckle tracking technique, whereas the analysis process is time-consuming and poorly reproducible. Chen et al. (26) used automated strain analysis techniques for the LA developed in parallel with related intelligent software, such as 4DLAQ, to accurately and rapidly obtain LA volumetric parameters, three-dimensional LA longitudinal strains, and LA circumferential strains. Moreover, Salte et al. (27) explored whether fully automated measurement techniques based on deep learning and AI are feasible and comparable to conventional speckle tracking applications for automated GLS when assessing LV function. The study found that this AI method was able to accurately classify all three standard apical views and successfully perform cardiac event timing in 89 percent of patients. In addition, the method successfully performed automated segmentation, motion estimation, and GLS measurements in all exams, eliminated measurement variability, and allowed for complete GLS analysis in less than 15 s. This suggests that fully automated AI-based measurements can facilitate the clinical practice of GLS.

#### 4.2.3 Automated valve analysis

As the population ages, the prevalence of heart valve disease is on the rise, posing a serious health and economic burden (28), and echocardiography has great advantages for the diagnosis of valve disease. Some scholars (10) used AI software to measure mitral valve geometry with parameters such as mitral annular area, annular height and width, leaflet conjunction spacing, and anterior and posterior leaflet length. It was found that better inter-observer agreement was obtained for all imaging parameters assessed and significantly less time was spent relative to conventional echocardiography. The AI software can also distinguish between organic and functional mitral regurgitation by automatically analyzing the anatomical and hemodynamic information of the mitral annulus, which can determine the cause of the disease to a certain extent (29). Transesophageal echocardiography is commonly utilized in clinical practice to evaluate valves, primarily because it offers the advantage of viewing the valves from multiple angles. However, this method can be complex, time-consuming, and often suffers from poor reproducibility. In response to these challenges, recent years have seen the development of AI software such as 4DLVQ, eSieValves, 4DautoMVQ, and 4D MV ASSESSMENT. These tools are designed to automatically analyze 3D ultrasound images of the valve annulus, dynamically track, and evaluate the generated valve images and their associated parameters. This technological advancement has demonstrated good feasibility, accuracy, and reproducibility in practice (30).

### 4.3 Research hotspots and directions

Current research hotspots in AI applications for echocardiography include image segmentation, automated cardiac function quantification, and disease assessment. For example, the EchoNet-Dynamic model developed by Professor Ouyang’s team (31). in 2020 demonstrated AI’s ability to assess LVEF comparably to sonographers, while also reducing cardiologists’ interpretation time. Similarly, Hathaway (32) et al. trained ML models on over 1,900 annotated cardiac ultrasound images to predict LV remodeling, achieving an area under the curve of 0.78 and 0.79 for point-of-care and high-end ultrasound, respectively. To improve clinical applicability, several strategies are essential. Transfer learning enhances model adaptability and generalizability in data-scarce settings (33), while explainable AI (XAI) can increase transparency by offering interpretable insights into AI decision-making, which is crucial for building clinical trust and acceptance (34). Regulatory compliance, including certification by the U.S. Food and Drug Administration (FDA) and the European Conformité Européenne (CE) marking, plays a crucial role in ensuring the safety and quality of AI applications. These processes are reinforced by standardized evaluation protocols that assess algorithmic bias and model performance (35).

In summary, AI is reshaping echocardiographic practice through improved accuracy and efficiency. Future work should focus on enhancing model generalizability, addressing ethical considerations, and establishing robust regulatory frameworks. Collaboration among developers, clinicians, and regulators will be key to ensure the safe and effective integration of AI into routine echocardiographic workflows.

### 4.4 Limitations

This study is subject to several limitations. Firstly, although the literature review spans from 2009 to 2024, there is a possibility that some publications from 2024 were not included due to their late release, potentially resulting in an incomplete analysis of the most recent trends. Secondly, the literature search was confined to the Web of Science Core Collection (WoSCC) SCI-Expanded database. While WoSCC is a widely recognized source, the exclusion of other major databases such as Scopus and PubMed may have led to the omission of relevant and regionally significant studies, including clinical preprints. To enhance comprehensiveness, future research should consider incorporating these databases. Thirdly, the analysis was restricted to English-language publications, which may have resulted in the omission of significant studies published in other languages, introducing a potential language bias. Thus, including non-English literature would help to reduce language bias and provide a more global perspective on AI applications in echocardiography.

In addition to the general limitations of bibliometric analyses, this study does not fully address the practical challenges faced by AI models in echocardiography. One significant issue is insufficient data diversity—for example, the underrepresentation of certain ethnic groups, age ranges, or comorbidities may lead to biased model performance and limited generalizability in real-world settings. AI tools primarily trained on adult data from high-resource regions may underperform in pediatric or underserved populations. According to an overview by the American College of Radiology, only 3% of FDA-approved AI applications for medical image analysis (7 out of 221 products) are specifically tailored for pediatric imaging (36). Another major concern is the “black-box” nature of many deep learning models, which lack interpretability and make it difficult for clinicians to understand how specific predictions are generated (37). This opacity can undermine clinical trust and hinder the adoption of AI systems. Moreover, ethical challenges—such as data privacy issues, lack of algorithmic transparency, and the risk of exacerbating healthcare disparities—also pose significant barriers. In echocardiography and broader medical practice, AI-generated results and feature extraction can directly influence clinical decisions, potentially altering patient management strategies (38).

To address these concerns, several strategies should be prioritized. These include multicenter data sharing to improve dataset diversity, the integration of XAI to enhance transparency and clinician trust, and prospective clinical validation to ensure real-world efficacy. Furthermore, aligning AI systems with regulatory standards such as FDA and CE certifications will be essential for safe and equitable implementation. Overall, these challenges warrant further investigation to support the robust, responsible, and widespread application of AI technologies in clinical echocardiography.

## 5 Conclusion

This study provides a comprehensive bibliometric analysis of AI applications in echocardiography from 2009 to 2024. Research output has surged since 2018, with China and the USA leading contributions. The Mayo Clinic stands out for its high impact and collaboration. Current focus areas include automated quantification of cardiac function and AI-driven myocardial strain and valve analysis. Future work should emphasize validating AI methods’ safety, efficacy, and generalizability to ensure clinical adoption. In sum, it is our aspiration that this investigative trajectory will garner increased scholarly interest and catalyze the development of innovative techniques, further embedding AI within the practice of echocardiography.

## Data Availability

The original contributions presented in the study are included in the article/Supplementary material, further inquiries can be directed to the corresponding authors.

## References

[1] Mitchell C Rahko PS Blauwet LA Canaday B Finstuen J Foster M . Guidelines for performing a comprehensive transthoracic echocardiographic examination in adults: recommendations from the American Society of Echocardiography. J Am Soc Echocardiogr. (2019) 32:1–64. doi: 10.1016/j.echo.2018.06.004 30282592

[2] Hagendorff A Stöbe S Helfen A Knebel F Altiok E Beckmann S . Echocardiographic assessment of atrial, ventricular, and valvular function in patients with atrial fibrillation-an expert proposal by the German working group of cardiovascular ultrasound. Clin Res Cardiol. (2025) 114:4–24. doi: 10.1007/s00392-024-02491-6 39186180 PMC11772422

[3] Fortuni F Ciliberti G De Chiara B Conte E Franchin L Musella F . Advancements and applications of artificial intelligence in cardiovascular imaging: a comprehensive review. Eur Heart J Imaging Methods Pract. (2024) 2:qyae136. doi: 10.1093/ehjimp/qyae136, PMID: 39776818 PMC11705385

[4] Chu WK Raeside DE. Fourier analysis of the echocardiogram. Phys Med Biol. (1978) 23:100–5. doi: 10.1088/0031-9155/23/1/009, PMID: 635004

[5] Zhang J Gajjala S Agrawal P Tison GH Hallock LA Beussink-Nelson L . Fully automated echocardiogram interpretation in clinical practice. Circulation. (2018) 138:1623–35. doi: 10.1161/CIRCULATIONAHA.118.034338, PMID: 30354459 PMC6200386

[6] Zhou J Du M Chang S Chen Z. Artificial intelligence in echocardiography: detection, functional evaluation, and disease diagnosis. Cardiovasc Ultrasound. (2021) 19:29. doi: 10.1186/s12947-021-00261-2, PMID: 34416899 PMC8379752

[7] Shen J Shen H Ke L Chen J Dang X Liu B . Knowledge mapping of immunotherapy for hepatocellular carcinoma: a bibliometric study. Front Immunol. (2022) 13:815575. doi: 10.3389/fimmu.2022.815575, PMID: 35173728 PMC8841606

[8] Ke L Lu C Shen R Lu T Ma B Hua Y. Knowledge mapping of drug-induced liver injury: a scientometric investigation (2010-2019). Front Pharmacol. (2020) 11:842. doi: 10.3389/fphar.2020.00842, PMID: 32581801 PMC7291871

[9] Sood SK Rawat KS Kumar D. Analytical mapping of information and communication technology in emerging infectious diseases using citespace. Telemat Inform. (2022) 69:101796. doi: 10.1016/j.tele.2022.101796, PMID: 35282387 PMC8901238

[10] Wang B Xing D Zhu Y Dong S Zhao B. The state of exosomes research: a global visualized analysis. Biomed Res Int. (2019) 2019:1495130. doi: 10.1155/2019/1495130 31073519 PMC6470441

[11] Yeung AWK Heinrich M Atanasov AG. Ethnopharmacology-a bibliometric analysis of a field of research meandering between medicine and food science? Front Pharmacol. (2018) 9:215. doi: 10.3389/fphar.2018.00215, PMID: 29599720 PMC5862826

[12] Sood SK Rawat KS. A scientometric analysis of ICT-assisted disaster management. Nat Hazards. (2021) 106:2863–81. doi: 10.1007/s11069-021-04512-3, PMID: 33500600 PMC7820517

[13] Wu H Tong L Wang Y Yan H Sun Z. Bibliometric analysis of global research trends on ultrasound microbubble: a quickly developing field. Front Pharmacol. (2021) 12:646626. doi: 10.3389/fphar.2021.646626, PMID: 33967783 PMC8101552

[14] Liu YX Yang Y Le KJ Zhang ZL Cui M Zhong H . Antimicrobial stewardship in surgery: a literature bibliometric analysis. Front Public Health. (2022) 10:847420. doi: 10.3389/fpubh.2022.847420, PMID: 35462840 PMC9021645

[15] Chen C Dubin R Kim MC. Emerging trends and new developments in regenerative medicine: a scientometric update (2000-2014). Expert Opin Biol Ther. (2014) 14:1295–317. doi: 10.1517/14712598.2014.920813, PMID: 25077605

[16] Zheng J Zhou R Meng B Li F Liu H Wu X. Knowledge framework and emerging trends in intracranial aneurysm magnetic resonance angiography: a scientometric analysis from 2004 to 2020. Quant Imaging Med Surg. (2021) 11:1854–69. doi: 10.21037/qims-20-729, PMID: 33936970 PMC8047370

[17] Kusunose K Haga A Abe T Sata M. Utilization of artificial intelligence in echocardiography. Circ J. (2019) 83:1623–9. doi: 10.1253/circj.CJ-19-0420, PMID: 31257314

[18] Alsharqi M Woodward WJ Mumith JA Markham DC Upton R Leeson P. Artificial intelligence and echocardiography. Echo Res Pract. (2018) 5:R115–r25. doi: 10.1530/ERP-18-0056, PMID: 30400053 PMC6280250

[19] Johnson KW Torres Soto J Glicksberg BS Shameer K Miotto R Ali M . Artificial intelligence in cardiology. J Am Coll Cardiol. (2018) 71:2668–79. doi: 10.1016/j.jacc.2018.03.521, PMID: 29880128

[20] Puttagunta M Ravi S. Medical image analysis based on deep learning approach. Multimed Tools Appl. (2021) 80:24365–98. doi: 10.1007/s11042-021-10707-4, PMID: 33841033 PMC8023554

[21] Sveric KM Ulbrich S Dindane Z Winkler A Botan R Mierke J . Improved assessment of left ventricular ejection fraction using artificial intelligence in echocardiography: a comparative analysis with cardiac magnetic resonance imaging. Int J Cardiol. (2024) 394:131383. doi: 10.1016/j.ijcard.2023.131383, PMID: 37757986

[22] Yang Y Lu M Guan X Zhao S Long L. Left atrial dysfunction in apical hypertrophic cardiomyopathy: assessed by cardiovascular magnetic resonance feature-tracking. J Thorac Imaging. (2024) 39:157–64. doi: 10.1097/RTI.0000000000000722, PMID: 37341629 PMC11027970

[23] Chang S Zeng D Zhang X Huang L Cai Y Huang T . Impact of non-valvular atrial fibrillation on mitral valve anatomic features: a study of three-dimensional mitral valve by transesophageal echocardiography and automatic analysis software. Echocardiography. (2024) 41:e15943. doi: 10.1111/echo.15943, PMID: 39387642

[24] Chang S Zhang X Ge C Zhong Y Zeng D Cai Y . Automatic echocardiographic assessment of left atrial function for prediction of low-voltage areas in non-valvular atrial fibrillation. Int J Gen Med. (2024) 17:4493–506. doi: 10.2147/IJGM.S477499, PMID: 39372132 PMC11456279

[25] Lang RM Badano LP Mor-Avi V Afilalo J Armstrong A Ernande L . Recommendations for cardiac chamber quantification by echocardiography in adults: an update from the American Society of Echocardiography and the European Association of Cardiovascular Imaging. J Am Soc Echocardiogr. (2015) 28:1–39. doi: 10.1016/j.echo.2014.10.003 25559473

[26] Chen L Zhang C Wang J Guo L Wang X Liu F . Left atrial strain measured by 4D auto laq echocardiography is significantly correlated with high risk of thromboembolism in patients with non-valvular atrial fibrillation. Quant Imaging Med Surg. (2021) 11:3920–31. doi: 10.21037/qims-20-1381, PMID: 34476178 PMC8339650

[27] Salte IM Østvik A Smistad E Melichova D Nguyen TM Karlsen S . Artificial intelligence for automatic measurement of left ventricular strain in echocardiography. JACC Cardiovasc Imaging. (2021) 14:1918–28. doi: 10.1016/j.jcmg.2021.04.018, PMID: 34147442

[28] Aluru JS Barsouk A Saginala K Rawla P Barsouk A. Valvular heart disease epidemiology. Med Sci. (2022) 10:32. doi: 10.3390/medsci10020032, PMID: 35736352 PMC9228968

[29] Armoundas AA Narayan SM Arnett DK Spector-Bagdady K Bennett DA Celi LA . Use of artificial intelligence in improving outcomes in heart disease: a scientific statement from the American Heart Association. Circulation. (2024) 149:e1028–50. doi: 10.1161/CIR.0000000000001201, PMID: 38415358 PMC11042786

[30] Garcia-Martin A Lazaro-Rivera C Fernandez-Golfin C Salido-Tahoces L Moya-Mur JL Jiménez-Nacher JJ . Accuracy and reproducibility of novel echocardiographic three-dimensional automated software for the assessment of the aortic root in candidates for thanscatheter aortic valve replacement. Eur Heart J Cardiovasc Imaging. (2016) 17:772–8. doi: 10.1093/ehjci/jev204, PMID: 26320167

[31] Ouyang D He B Ghorbani A Yuan N Ebinger J Langlotz CP . Video-based AI for beat-to-beat assessment of cardiac function. Nature. (2020) 580:252–6. doi: 10.1038/s41586-020-2145-8, PMID: 32269341 PMC8979576

[32] Hathaway QA Yanamala N Siva NK Adjeroh DA Hollander JM Sengupta PP. Ultrasonic texture features for assessing cardiac remodeling and dysfunction. J Am Coll Cardiol. (2022) 80:2187–201. doi: 10.1016/j.jacc.2022.09.036, PMID: 36456049

[33] Ma K Shen C Xu Z He D. Transfer learning framework for streamflow prediction in large-scale transboundary catchments: sensitivity analysis and applicability in data-scarce basins. J Geogr Sci. (2024) 34:963–84. doi: 10.1007/s11442-024-2235-x

[34] Bienefeld N Boss JM Luthy R Brodbeck D Azzati J Blaser M . Solving the explainable AI conundrum by bridging clinicians' needs and developers' goals [J]. NPJ Digit Med. (2023) 6:94. doi: 10.1038/s41746-023-00837-4, PMID: 37217779 PMC10202353

[35] Pemberton JS Wilmot EG Barnard-Kelly K Leelarathna L Oliver N Randell T . CGM accuracy: contrasting CE marking with the governmental controls of the USA (FDA) and Australia (TGA): a narrative review. Diabetes Obes Metab. (2023) 25:916–39. doi: 10.1111/dom.14962, PMID: 36585365

[36] Singh SB Sarrami AH Gatidis S Varniab ZS Chaudhari A Daldrup-Link HE. Applications of artificial intelligence for pediatric cancer imaging. AJR Am J Roentgenol. (2024) 223:e2431076. doi: 10.2214/AJR.24.31076, PMID: 38809123 PMC11874589

[37] Petch J Di S Nelson W. Opening the black box: the promise and limitations of explainable machine learning in cardiology. Can J Cardiol. (2022) 38:204–13. doi: 10.1016/j.cjca.2021.09.004, PMID: 34534619

[38] Aziz D Maganti K Yanamala N Sengupta P. The role of artificial intelligence in echocardiography: a clinical update. Curr Cardiol Rep. (2023) 25:1897–907. doi: 10.1007/s11886-023-02005-2, PMID: 38091196

