# Supplementary material for: Application of artificial intelligence in echocardiography from 2009 to 2024: a bibliometric analysis
Source: Front Med (Lausanne). 2025 Jul 29;12:1587364. doi: 10.3389/fmed.2025.1587364 (PMC12339534; doi:10.3389/fmed.2025.1587364)
Supplement: Supplementary file 1 [file Table_1.DOCX]

**Table S1.** The 10 most citations co-cited authors.

| Rank | Author | Institution (country) | Citations | TLS |
| --- | --- | --- | --- | --- |
| 1 | He, Kangmin | University of Chinese Academy of Sciences (China) | 411 | 5545 |
| 2 | Lang,R.M | University of Chicago (USa) | 369 | 4249 |
| 3 | Ronneberger, Olaf | University of Freiburg (Germany) | 330 | 4489 |
| 4 | Acharya, U. Rajendra | University of Southern Queensland (Australia) | 295 | 3941 |
| 5 | Zhang,J | Donghua University (China) | 278 | 4292 |
| 6 | Attia, Itzhak Zachi | Mayo Clinic (USA) | 217 | 2334 |
| 7 | Simonyan, Kristina | Harvard University (USA) | 206 | 3080 |
| 8 | Christian Szegedy | Cadence Berkeley Labs (USA) | 199 | 3325 |
| 9 | LeCun, Yann | China Jiliang University (China) | 194 | 2805 |
| 10 | Breiman,L | University of California Berkeley (USA) | 185 | 1295 |

TLS: total link strength.

**Table S2.** The 10 most co-cited journals.

| Rank | Journal | Documents | Citations | TLS | Impact factor (2024) | JCR |
| --- | --- | --- | --- | --- | --- | --- |
| 1 | Ultrasound in Medicine and Biology | 89 | 1248 | 257 | 2.4 | Q2 |
| 2 | Diagnostics | 88 | 435 | 261 | 3 | Q1 |
| 3 | Frontiers in Cardiovascular Medicine | 85 | 445 | 260 | 2.8 | Q2 |
| 4 | Scientific Reports | 77 | 515 | 175 | 3.8 | Q1 |
| 5 | Ieee Access | 58 | 357 | 179 | 3.4 | Q2 |
| 6 | European Radiology | 54 | 1385 | 188 | 4.7 | Q1 |
| 7 | Journal of Clinical Medicine | 52 | 423 | 247 | 3 | Q1 |
| 8 | PLoS One | 50 | 701 | 94 | 2.9 | Q1 |
| 9 | Medical Image Analysis | 49 | 1402 | 273 | 10.7 | Q1 |
| 10 | Computers in Biology and Medicine | 49 | 873 | 179 | 7 | Q1 |

TLS: total link strength.
